# King George's Visit to the London Hospital

**Published:** 1910-08-06

**Authors:** 


### KING GEORGE'S VISIT TO THE LONDON HOSPITAL.

By the express wish of his Majesty, arrangements were made last Saturday for him and the Queen to visit the London Hospital. No happier proof of King George's interest in our hospitals could have been given than this spontaneous wish of his to inspect—and, as is proved from the fact that an hour was spent instead of the thirty minutes given in the programme, to inspect most carefully—the great hospital of the East End.

By good fortune it was a fine, sunny afternoon when the Royal carriage with its escort of Household Cavalry drove up to the main entrance. Their Majesties were received by the Earl of Derby, treasurer of the hospital, and the Hon. Sydney Holland, chairman, who conducted them through the hall to the garden, where the house committee and other officers were presented, Mr. Morris, secretary and author, among them at the King's request.

### The Scene in the Quadrangle.

More than one interesting fact robbed the presentations of mere formality. Thus, when Mr. Morris was presented, his Majesty reminded those present that some years ago he had presented Mr. Morris with Sir Edgar Speyer's prize for an essay on "Hospital Management." Sir Frederick Young, who, with Mr. Eve (senior surgeon), Sir Frederick Treves, Dr. Warner (senior physician), Dr. Dawson, and Mr. Rigby, was also presented, is the oldest vice-president of the hospital, being now ninety-four years of age. Mr. Hora, the permanent benefactor of the institution by his gift of the Marie Celeste Ward, was similarly honoured. Last, but not least, Miss McIntosh, the newly appointed matron at St. Bartholomew's Hospital, who, after the formal presentation was over, was recalled by the King and Queen for a few private words, completed the list of those who received special distinction.

The garden, which looked delightful in the bright sun, was made still brighter by the blue uniforms of the sisters and the red blankets of the patients, many of whom were on the balconies which run round the quadrangle. In the centre of the latter stands the fine statue of Queen Alexandra, to which their Majesties paid special attention, and the King, on seeing the afore-mentioned groups on the balconies, remarked how beneficial these are to patients, and how much he wished that all hospitals could be provided with them.

After crossing the quadrangle the Royal party, which included Princess Mary, walked on to the grass tennis court beyond, where many convalescent patients were seated, with a sprinkling of students among them. To more than one of the patients their Majesties addressed a few words, and we may remark here that among these cases, in which, medically as well as humanly speaking, King George took an interest was one of leprosy. This, of course, is a rare case to see in England, and not a less rare sight at the London, the one in question being temporarily received there (as not being an infectious disease) till the Local Government Board can deal with it. His Majesty, however, in the course of his travels has visited Robben Island, and it was therefore not the strangeness of the disease to him which awoke his sympathy and interest.

### Points in the Inspection.

On leaving the tennis court and garden the King and Queen, amidst hearty cheering, made their way under an awning to the ground floor, where much time was spent in inspecting, and where the Queen most graciously presented prizes to the three fortunate probationers—Miss McNabb, Miss Derrick and Miss Reed—who took the highest places in the recent examinations. This presentation afforded another example of his Majesty's alert interest in the inspection. He remarked to the prize-winners that while no one could underrate the importance of efficiency in their work, there was another quality which still more he valued, and which he was glad to hear they possessed—the quality of human sympathy, without which the best work was but half done.

A visit was then paid to the Finsen-light department, reached by lift from the ground floor. Here the treatment for lupus is carried out, and the King had explained to him its details. The *x*-ray department was also examined, particularly the four rooms invented by Dr. Sequeira, by which the operators are protected from the risk of injury. His Majesty was most interested in these, for he did not forget the unfortunate accidents which have attended *x*-ray work in the sad cases of the late Mr. Cox and Mr. Harnack only lately. The new process by which oxygen is administered, by means of the oxygen-bed as invented by Dr. Leonard Hill, was also explained to their Majesties, one of its advantages being that the waste which attended the old system is avoided.

### The King's Previous Visit.

One of the features of the inspection which will strike the general, and even the hospital, world most, is the fact that the King showed himself no stranger to the building. When the out-patient department was reached his Majesty remarked that he knew his way about it without guidance. That he did so is proved from the fact that, apart from the visits which he paid officially when Prince of Wales, on one occasion a complaint of certain conditions led him to pay a private and unexpected visit, when he satisfied himself of the state of affairs and of the baselessness of the untoward rumour which had reached him. This visit had remained a secret to everyone except two at the hospital till Saturday last, when the King astonished everyone by relating it.

The above incidents of the King's visit are but a selection of many others which might be given did space permit, and are infinitely valuable as proving beyond doubt that a Royal inspection is a real inspection, and an inspection by an expert who knows where lie the real virtues and where the true faults beneath the surface that is presented to him. The London Hospital may be congratulated on having been the first not of hospitals only, but of all social institutions in the country, to receive the Royal attention, and there is no doubt that it has come out of the inspection with its reputation as high as ever and its efficiency consolidated by the knowledge that its aims and equipment are known to King George, who has proved by his personal examination his appreciation and understanding of them both.

### The King and the Crowds.

Two further facts, however, remain to be told. The first was his Majesty's farewell to Miss Luckes, the matron, while Miss McIntosh had the honour of conducting Princess Mary through the children's wards, while the King and Queen were inspecting the patients in the lupus ward, thus giving to her a unique memory to carry away from the London when she takes up her new duties at Bart.'s, a memory which brings to a fitting close her connection with the sister institution. The second was his Majesty's consideration for the crowds outside in the street. As we have said.

already, the inspection was a long one, and when it was debated whether to extend it by the tantalising inducement of visiting yet one ward more, the King caught sight of the people waiting him in the roadway, and said he could not keep them any longer beyond the time he was expected to leave.

Thus closed a visit which the London Hospital will remember as one of the most gracious and most useful it has ever received from Royalty. We congratulate it on a day that from first to last was a success, and a success well deserved from the years of patient hard work which alone have made it possible. The moral of it all is that King George has been at special pains to prove that he is following the example of King Edward in insisting on the highest standard of hospital administration. The hospitals must therefore do their part to carry on the great tradition established in Queen Victoria's reign, encouraged by the keen interest in their welfare which has never flagged in the Royal Family, and of which Saturday's inspection will long remain the most telling example.

